# Diagnosing knee osteoarthritis in patients, differences between general practitioners and orthopedic surgeons: a retrospective cohort study

**DOI:** 10.1186/s12875-023-02107-4

**Published:** 2023-08-01

**Authors:** Bob J. Evers, Henk J. Schers, Cornelia H. M. van den Ende, Rogier M. Thurlings, Sander Koëter

**Affiliations:** 1 Radboud Institute for Molecular Life Sciences, Radboud University Medical Centre, Nijmegen, Netherlands; 2 Department of Primary and Community Care, Radboud University Medical Centre, Nijmegen, The Netherlands; 3 Department of Rheumatic Diseases, Radboud University Medical Center, Nijmegen, The Netherlands; 4 Department of Orthopedics, Canisius Wilhelmina Hospital, Nijmegen, The Netherlands

**Keywords:** Primary care, Knee complaints, Osteoarthritis, Referrals, General practitioners, Orthopedic surgery

## Abstract

**Background:**

knee complaints are one of the most common reasons to consult the general practitioners in the Netherlands and contribute to the increasing burden on general practitioners. A proportion of patients that are referred to orthopedic outpatient clinics are potentially referred unnecessarily. We believe osteoarthritis is not always considered by general practitioners as the cause of atraumatic knee complaints. This may impede early recognition and timely care of osteoarthritis complaints and lead to unnecessary referrals.

**Methods:**

the aim of this study was to compare the frequency of (differential) diagnosis of osteoarthritis mentioned in referral letters of general practitioners with the frequency of osteoarthritis mentioned as orthopedic diagnosis at the outpatient clinic. Therefore we conducted a retrospective cohort study based on data collected from referral letters and the corresponding outpatient clinic reports of patients with atraumatic knee complaints of 45 years or older referred to a regional hospital in Nijmegen, The Netherlands in the period from 1-6-2019 until 1-01-2020.

**Results:**

a total of 292 referral letters were included. In the younger aged patients (45–54 years) osteoarthritis was mentioned less frequent and meniscal lesions were mentioned more frequent in referral letters when compared to diagnoses made at the outpatient clinic. Differences in differential diagnosis of osteoarthritis as well as meniscal lesions between orthopedic surgeon and general practitioners were found (both p < 0.001, McNemar). Matching diagnoses were present in 58.2% when all referral letters were analyzed (n = 292) and 75.2% when only referrals containing a differential diagnosis were analyzed (n = 226). Matching diagnoses were present in 31.6% in the younger age categories (45–54 years). A linear trend showing fewer matching diagnoses in younger patient categories was observed (p < 0.001).

**Conclusions:**

Osteoarthritis was less frequently mentioned in general practitioner referral letters among the differential diagnosis then it was diagnosed at the outpatient clinic, especially in younger patients (45–54 years). Also matching diagnoses in younger patients were evidently lower than in older patients, partly explained by underdiagnosing of osteoarthritis in younger patients in this cohort. Better recognition of osteoarthritis in younger patients and changing the diagnostic approach of general practitioners might improve efficacy in knee care. Future research should focus on the effectiveness of musculoskeletal triage, the need for multidisciplinary educational programs for patients and promotion of conservative treatment modalities among general practitioners.

## Introduction

Most consultations in general practice (GP) in the Netherlands are related to the musculoskeletal system [1]. Due to the ageing population in the Netherlands, the prevalence of musculoskeletal disorders in general and osteoarthritis (OA) in specific will rise in the future as OA is expected to be the most common medical condition in the Netherlands by 2040 [2]. Among musculoskeletal disorders knee problems stand out as the most common reason for consulting the GP as the incidence of knee complaints is currently 35.2 per 1000 patients years [1]. After the age of 45 the incidence of knee OA rises sharply with increasing age, making it the most prevalent diagnosis leading to disability of posture and movement worldwide [3, 4]. This expected increase will cause a growing burden on the health care system making careful triage of patients with knee complaints in primary care desirable to ensure that patients receive optimal care and the healthcare system remains sustainable.

Optimal care includes step-up treatment as proposed in the stepped care strategy (SCS) in which non-surgical treatment options such as physiotherapy, pain medication, diet advice, and surgical care in a structured and timely manner [5, 6]. In the Dutch healthcare system, the GP acts as a gatekeeper to hospital and specialist care and decides which patients with knee complaints to refer to hospital specialist care or when to refer to other healthcare professionals such as physical therapists or dietitians [7].

However, referring patients with knee complaints to orthopedic outpatient clinics (OPC) can be challenging as previous studies suggest that one third of all referrals to OPS’s are inappropriate because patients were either referred too early, for example because (in hindsight) more adequate treatment could have been given by the GP or referrals should have been to other medical specialties [8, 9]. Furthermore, recent reviews have stressed that OA is not limited to older age groups and that acknowledging the existence of OA in younger individuals is important [10, 11].

Early recognition of OA is required to ensure structured and timely usage of treatment modalities that are part of the SCS [6]. This strategy can improve non-surgical treatment and may prevent untimely referral and consequent surgery [6, 12]. To our knowledge no data on referral patterns to Dutch medical specialists for knee complaints is available.

We believe that OA is not always recognized as cause of complaints in patients referred with atraumatic knee complaints to the orthopedic OPC. This could lead to untimely and inappropriate referrals. Therefore we conducted a cohort study to describe and compare (differential) diagnoses in GP referrals for non-traumatic knee complaints with diagnosis made at the orthopedic OPC and to describe the extent of matching diagnoses with emphasis on knee OA.

## Methods

In this cohort study, we compared the referral (differential) diagnosis made by the GP and orthopedic diagnosis made at the OPC. The diagnosis made at the OPC was considered a gold standard.

### Study population

Data of patients referred by GPs to the OPC in a general hospital in Nijmegen for atraumatic knee complaints in the period between 1-6-2019 until 1-01-2020 was analyzed as this was the most recent period of 6 months before measures were taken to control the spread of SARS-CoV-2. All consecutive patients with a referral to an orthopedic surgeon (OS) for a first episode of knee complaints and aged 45 years and older were included. The age range was based on the Dutch GP guideline for atraumatic knee complaints [13]. Exclusion criteria were previous knee surgery or complaints that were attributed to recent trauma in referral letter or in the orthopedic medical record.

### Data collection

Data was extracted from both referral letters and corresponding medical records from the orthopedic OPC. The following referral characteristics were recorded from each referral letter: age, gender, the duration of knee complaints prior to referral, referral diagnoses and, if present, the treatment proposition. We categorized mentioned treatment proposals in referral letters as either proposing a therapeutic treatment proposal (e.g.surgery or intra articular injection) or a diagnostic treatment proposal (e.g. analysis of knee complaints, assessment of knee complaints). Data extracted from the reports of the OPC appointment included: diagnosis as mentioned by the OS (orthopedic surgeon) in the OPC report, additional diagnostic procedures ordered, and the proposed surgical (e.g. total or unilateral knee replacement surgery or arthroscopic surgery) or non-surgical (e.g. physical therapy or intra-articular injection) treatment options. Conventional radiography was not considered an additional diagnostic procedure, since local protocol required a full weight bearing conventional knee radiograph in all patients referred for chronic knee complaints. The severity of radiological OA progression was graded according to Kellgren and Lawrence on a scale ranging from 0 to 4 [14]. All conventional knee radiographs were scored by researchers BE and SK. Cases with no initial agreement were discussed until consensus was reached. For further analyses, patients were categorized in age groups: 45–54, 55–64, 65–74, 75–84 and over 85 years old. Data collection was performed by BE .

### Study outcomes

The primary outcome was the difference between differential diagnoses in GP referral letters and the orthopedic diagnosis at the OPC. To further illustrate the possible differences we determined matching diagnoses in referral differential diagnosis and orthopedic diagnosis made at the OPC.

Secondary outcomes were: [1] the distribution of treatment applied by the OS at the OPC: surgical (total or unilateral knee replacement surgery, arthroscopic surgery), conservative (education, oral pain medication, referral to dietician or physical therapy, intra-articular injection or physical therapy) among referred patients, [2] the description of patients referred with the request for surgery and the incidence of performed surgeries, [3] the description of radiological OA and incidence of surgical treatment, [4] the description of additional diagnostic procedures performed by the OS in patients referred for meniscal lesions and arthroscopic surgery rates, [5] description of duration of complaints prior to referral and surgical treatment, [6] description of the application of SCS is mentioned in referral letters and the prescription of treatment modalities described in the SCS at the OPC.

### Statistical analysis

Data analysis was performed using SPSS (version 24) and p-values lower than 0.05 were considered significant. Baseline descriptive statistics were calculated as mean and standard deviation (SD) or numbers with percentages. Matches in diagnosis was scored “present” when the diagnosis made by the OS was also mentioned in the (differential) diagnosis in the referral letter of the GP. The McNemar test was used to determine statistically significant differences between proportions of OA as well as meniscal lesions diagnoses between the GP and the OS. The chi-squared test for association was used to determine linear association between age categories and matching diagnosis. Differences in incidence of surgical treatment was calculated by using the Chi-squared test statistics. Differences in mean K&L scores between surgically treated and conservatively treated patients was calculated by using t-test statistics.

### Sample size calculation

All eligible patients between 1-6-2019 and 1-1-2020 were included. Because there was no previous availiable data on this subject, a sample size of convienence was chosen. We aimed to include at least 250 patients.

## Results

### Referral characteristics

**Fig. 1:**
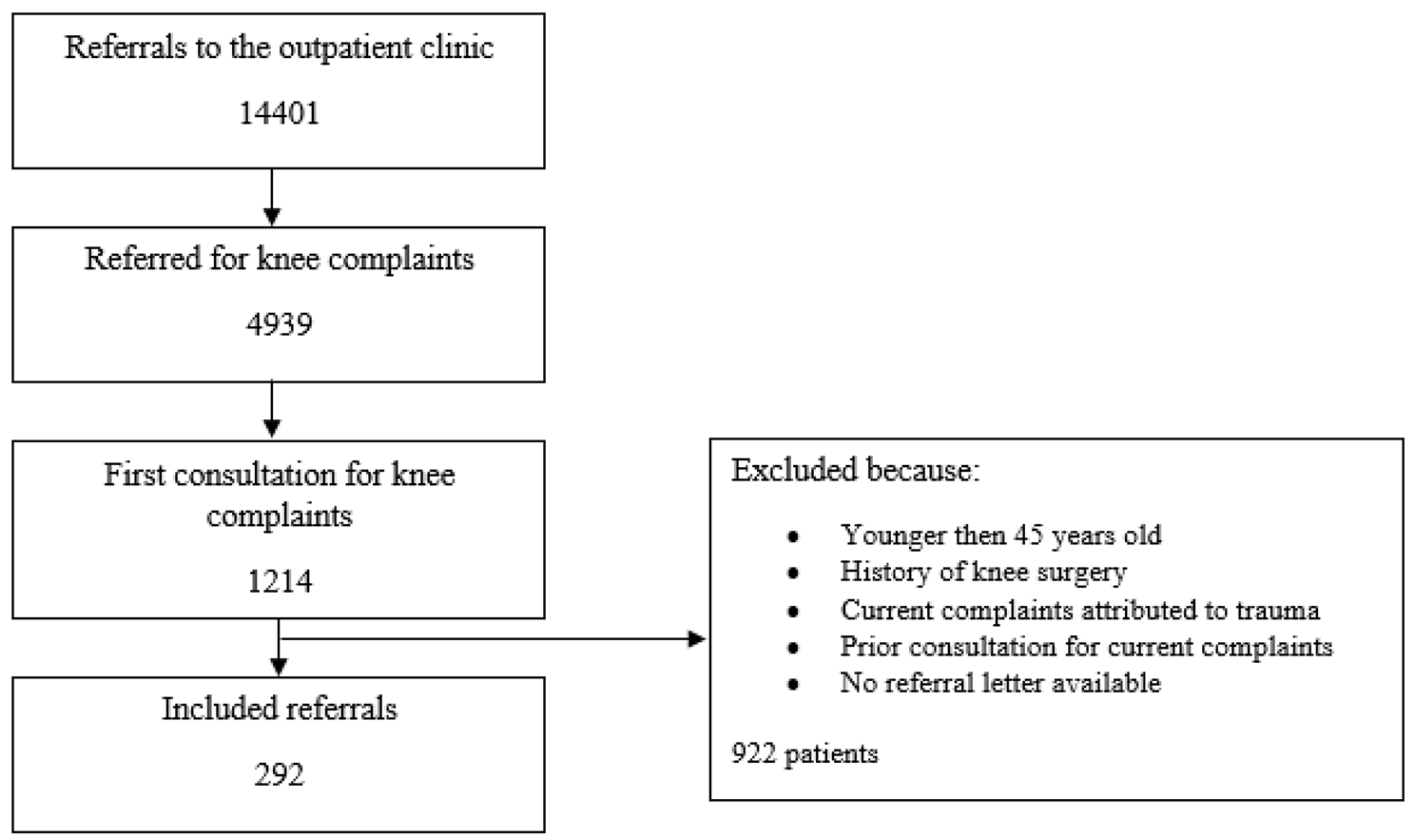
Flowchart showing the inclusion of referrals to the orthopedic outpatient clinic

A total of 292 patients were included (Fig. 1).

The mean (SD) age of referred patients was 65.0 (± 10.7) years. The majority of patients were female (N = 169; 57.9%). A referral diagnosis was mentioned in 226 referral letters (77.4%) and no diagnosis was mentioned in 66 referral letters (22.6%). Referral diagnoses consisting of 2 or more diagnoses were present in 50 referral letters (17.1%). Thirty-two patients (11.0%) were diagnosed with more than one diagnosis at the OPC.

At least one treatment proposal was mentioned in most of referrals letters (N = 216, 74%,).

When categorized in either therapeutic or diagnostic treatment proposals, therapeutic treatment proposals were present in 167 (57.2%), diagnostic treatment proposals were present in 48 (16.4%) in 77 (26.4%) patients no treatment proposal was present. Surgery was proposed in 40 referrals (13.7%), intra-articular injection was proposed in 10 referrals (3,4%).

### Primary outcomes

The most frequently mentioned referral differential diagnosis was OA (N = 152, 55.3%) followed by meniscal lesions (N = 77, 28%). The most frequent diagnosis by the OS was OA (N = 229, 69.6%), followed by meniscal lesions (N = 47, 14.3%). With respect to OA diagnosis, agreement was reached in 178 patients (33 patients without OA, 145 patient with OA). Forty-one patients that received the diagnosis OA by the OS were not referred for OA by the GP. Seven patients that did not receive the diagnosis OA by the OS where referred for OA by the GP. With respect to meniscal lesions, agreement was reached in 171 patients (144 without meniscal lesion, 27 patients with meniscal lesions). Nine patients that received the diagnosis meniscal lesion by the OS where not referred for meniscal lesion by the GP. Fifty patients that did not receive the diagnosis meniscal lesion by the OS were referred for meniscal lesions by the GP. The difference in both these paired proportions was statistically significant (p = < 0.001, McNemar).

While the percentages of patients referred with referral diagnosis OA increased with age, referrals for meniscal leasion decreased with rising age. The same trend is observed in diagnoses made at the OPC, but the percentage of OA diagnoses at the OPC was higher and the percentage of meniscal lesion was lower in all age groups compared to the referral diagnoses (Table 1).

**Table 1:** Diagnoses per age category

	Diagnosis	45–54 N (%)	55–64 N (%)	65–74 N (%)	75–84 N (%)	> 85 N (%)	Total N
Referral diagnosis by GP*	osteoartritis	6 (12.5)	45 (51.7)	55 (64.7)	39 (83.0)	7 (87.5)	152
	meniscal lesion	27 (56.2)	24 (27.5)	20 (23.5)	6 (12.8)	0 (0.0)	77
	other diagnosis***	15 (31.2)	18 (20.6)	10 (11.8)	3 (4.2)	1 (12.5)	46
Outpatient clinic diagnosis by OS**	osteoarthritis	21 (32.8)	73 (69.5)	78 (82.1)	47 (90.6)	10 (83.3)	229
	meniscal lesion	23 (35.9)	15 (14.2)	7 (7.4)	2 (3.8)	0 (0.0)	47
	other diagnosis****	20 (31.3)	17 (16.1)	10 (10.5)	4 (7.5)	2 (16.7)	53

*General practitioner

**Orthopedic surgeon

***other diagnosis include: anterior knee pain, cruciate ligament lesion, collateral ligament lesion, tendinopathies/myalgia, loose body, osteochondral lesion,, chondrocalcinosis,, Schwanoma, joint overuse, psoriatic arthritis, exostosis or findings secondary to intra-articular disease (e.g. Bake’s cyst, joint effusion)

****other diagnosis include:Anterior knee pain, intra-articular infection, cruciate ligament lesion, collateral ligament lesion, tendinopathies/myalgia, loose body, bone marrow edema, pigmented villonodular synovitis, osteochondral lesion, gout/rheumatic disease, chondrocalcinosis, Bakers cyst, Schwanoma,, exostosis, iliotibial band syndrome, hernia nucleus pulposus

Matches between one of the referral diagnoses and one of the orthopedic diagnosis were present in 170 (58.2%) referrals. Of referrals that contained a referral diagnosis (N = 226), 75.2% (N = 170) showed a match between referral diagnosis and orthopedic diagnosis. Matches in the referral diagnosis were lower in the category of younger patients, with the lowest percentage of 21% (N = 12) in the age category 45–54 years. A statistically significant linear association between age category and the proportion of matching diagnosis was present(Table 2).

**Table 2:** Matching diagnosis per age category

Age category	Matching diagnosis of referrals with DD** present only N = 226,%* (N)	
45–54	31,6 (12)	
55–64	73,6 (53)	
65–74	85,5 (59)	
75–85	97,5 (39)	
> 85	100 (7)	
P value for linear-by-linear association	(< 0.001)	

*Percentage of referrals with a matching diagnosis in corresponding age group

**Differential diagnosis

OA was the most prevalent diagnosis at the OPC in referrals where there was no match of the diagnosis in the age category of 45–54 years (N = 19, 43%).

### Secondary outcomes

Within patients that received surgical intervention (N = 87, 29.8%,), total knee replacement was the most frequent performed surgery (N = 57) after referral, followed by unilateral knee replacement (N = 15) and arthroscopy surgery (N = 13). Surgery rates were highest in patients aged 75–85 years. Total knee replacement was performed more frequent in older age categories compared to younger age categories. Unilateral knee replacement was performed less often in older age categories and arthroscopic surgery was performed in the two youngest age categories only (Table 3).

**Table 3:** Surgery rate per age category

Age category	Patients that received surgery N,(%*)	Total knee replacement, N (%**)	Unilateral knee replacement, N (%**)	Arthroscopic knee surgery, N (%**)	Removal of bone fragment, N (%**)	Excision of Schwannoma, N (%**)
45–54	13 (23,2)	2 (15,4)	1 (7,7)	10 (76,9)	1 (100,0)	0 (0)
55–64	25 (26,6)	15 (60,0)	7 (28)	3 (12)	0 (0)	0 (0)
65–74	21 (25,6)	16 (76,2)	5 (23,8)	0 (0)	0 (0)	1 (100,0)
75–85	24 (50,0)	22 (91,7)	2 (8,3)	0 (0)	0 (0)	0 (0)
> 85	2 (16,7)	2 (100,0)	0 (0)	0 (0)	0 (0)	0 (0)

*Percentage of surgery in corresponding age category

**Percentage within patients that received surgery

Of the 74 (25.3%) patients that were referred with meniscal lesions being among the differential diagnoses, arthroscopic surgery was performed in 8 (10.8%) patients. Among conservatively treated patients, “patient education” was the most prevalent treatment modality (N = 139). Forty-seven referred patients (16.1%) received no other treatment modality than “education” at the OPC. Additional steps from the SCS were applied in 162 patients (55.4%): intra-articular injection was proposed to 92 patients (31.5%), referral to physiotherapy in addition to education was advised to 70 (23.9%) patients. No medication was prescribed, and none of the patients were referred to a dietician by the OS, but weight loss advice was included in the education module at the OPC.

The incidence of surgical treatment was significantly higher in patients that were referred with a request for surgery than in patients without a request for surgery in their referral letter (62.5% VS 24.6%) (χ² (1, N = 292) = 0.00, p = 0.00, phi = 0.285)).

The mean K&L score of referred patients was 2.15 (SD; 1.29). K&L score was significantly higher in patients that were treated with total knee or unilateral joint replacement surgery when compared to conservatively treated patients (3.54 (SD; 0,58) vs. 1.69 (SD 1.12); P < 0.001).

MRI as a diagnostic procedure to identify meniscal lesion was requested for 42 patients (14.4%). Among patients that received an MRI-scan, “meniscal lesion” was most commonly mentioned in referral diagnoses (N = 20) followed by OA (N = 7). In more than half of patients that received an MRI-scan, a meniscal lesion was present (N = 23, 53.5%). In 3 patients (1.0%) a result of a MRI-scan was already at the disposal of the GP before referral to the OS. Matching diagnosis was present in all 3 patients.

When categorized for duration of complaints prior to referral, a duration of 3 months until one year was most common (N = 115; 39.3%) followed by a duration of complaints longer than 1 year (N = 112; 38.3%). With increasing duration of complaints, OA became a more prevalent referral diagnoses. The opposite was observed for referrals for mensical laesions (Table 4), which became less prevalent with increasing duration of complaints.

**Table 4:** Diagnoses per duration of complaints

Referral diagnosis	0–3 months, N (%)	3–12 months, N (%)	> 12 months, N (%)
No diagnosis	13 (16,7)	30 (21,9)	20 (15,9)
Osteoarthritis	21 (26,9)	51 (37,2)	77 (61,1)
Meniscal lesion	28 (35,9)	28 (27,7)	13 (10,3)
Other diagnosis*	16 (20,5)	28 (20,4)	16 (12,7)

*Other diagnoses include: patella femoral pain syndrome, cruciate ligament lesion, collateral ligament lesion, tendinopathies/myalgia, loose body, osteochondral lesion, effusion, chondrocalcinosis, Bakers cyst, Schwanoma, joint overuse, psoriatic arthritis, exostosis, patella maltracking

In the referral letters a history of treatment with physiotherapy (N = 32), intra-articular injections (N = 26), oral pain medication (N = 16), weight reduction (N = 3), or (non specified) conservative therapy (N = 3) was mentioned. History of applied steps for the SCS were mentioned in 38 (13%) referral letters of patients referred for OA. Treatment steps described in SCS were prescribed in 70 (31.3%) patients in whom OA was among the differential diagnoses.

## Discussion

The current study showed that there are differences in diagnoses made by GP’s and at the OPC in patients over 45 years old referred for atraumatic knee complaints. While OA was mentioned less frequently in referral letters by GP’s, meniscal lesions were mentioned more frequently, especially in younger patients (aged 45–54 years). Apart from differences in rates of diagnosis of OA and meniscal lesions observed in younger patients, differences in the rates of matching diagnoses were also observed, with higher rates of matching diagnoses observed in older patients groups when compared to younger patients groups. The higher rates of matching diagnoses in older patient categories can be explained by the increasing prevalence of OA in older patients in the current study, which is in line with increasing proportion of OA in older patients observed in epidemiological studies. The lower rates of matching diagnoses in younger patients may be explained by the fact that OA was less frequently recognized as cause of complaints or reflect a reluctance of GPs to diagnose a chronic illnesses in younger patients. In the current study OA was underdiagnosed. This is supported by the fact that in our data OA was the most prevalent diagnosis made at the OPC in the patients with a mismatch between referral diagnosis and OPC diagnosis. These patients could potentially have received a suboptimal conservative treatment in general practice (not based on SCS) prior to referral as treatment could have been based on an inappropriate diagnosis.

Surgery was advised as treatment in approximately one third of the referrals at the OPC. However, referrals that contained a request for surgery by the GP were given a surgical treatment significantly more often. This indicates that GP’s assess the need for surgery reasonably well.

OA became the more prevalent referral diagnosis in patients that had a longer duration of complaints prior to referral.

Previous studies have reported that 27–43% of referrals to orthopedic OPC were inappropriate [8, 9]. The rates of mismatching referral diagnosis might be similar in our study, but the current design is not suitable for making statements on the appropriateness of the referrals. The appropriateness of the referral is not limited to the rate of surgically treated patients or matching diagnosis, as referral to non-surgical secondary care and a consultation on request of a patient for example may also be considered appropriate care. Surgery rates found in the current study are in line with those found in previous studies [15, 16], which reported a surgery rate of 31–34% of patients referred to secondary care. Discrepancy with other studies such as McHugh [17], who reported a surgery rate of 50% is possibly explained by the nature of that study population, since the McHugh only included patients referred to OSs for consideration of total joint replacement.

Considering the current data, there seems to be room for improvement. Optimizing care for atraumatic knee complaints is preferable as it might improve the patients burden and decrease health care utilization by lowering potentially unnecessary referral and untimely surgery [12]. Furthermore, as the majority of patients referred with meniscal lesions are treated conservatively, these patients might have been treated in general practice exclusively, potentially preventing unnecessary referrals. This is in line with the changing perspective on optimal care for meniscal lesion reflected in the 2016 ESSKA meniscus consensus, stating that arthroscopic partial meniscectomy should not be proposed as a first-line treatment in patients with a degenerative meniscus lesions. This study seems to reflect the specific diagnostic dilemma in younger patients.

To improve the of care for atraumatic knee complaints certain aspects of the referral process might need further evaluation. First off, previous studies indicated a low level of confidence among GP’s in their abilities to diagnose musculoskeletal disorders [18, 19]. Changing beliefs on optimal treatment procedures among OSs might take time to be fully adopted by GPs and therefore additional training programs might be of value. Unfortunately, to our knowledge no current literature on this topic exists. Furthermore, recent systematic reviews have shown that musculoskeletal triage by professionals, such as extended scope physiotherapists, can possibly reduce healthcare utilization and help manage GP workload [20–22].

Another factor that contributes to potentially unnecessary referrals might be that some patients have a strong preference for referral to a specialist [23]. Multidisciplinary educational programs on degenerative knee complaints in primary care might assist in gaining patient trust, improving selfcare and adjust health care seeking behavior [12].

Finally a history of applied steps from the SCS were mentioned in only a quarter of referral letters with OA among the differential diagnosis. This might be due to the fact that not all applied treatment steps were mentioned in the referral letters. However, other studies have reported the underuse of conservative treatment modalities in knee OA [24–26]. In our data, 70 patients (46%) with OA in the referral diagnosis were treated with conservative treatment modalities at the OPC. Improved application of SCS in general practice can therefore potentially prevent unnecessary referrals.

Although the present study was the first to research referral patterns for atraumatic knee complaints in a general hospital in Netherlands, it also has limitations. A first limitation is that extrapolation of these data to different countries should be done with caution because settings differ, as well as healthcare systems and access to care across countries. For instance, in the Netherlands the GP acts as a gatekeeper for referral to secondary care services such as an OS, whereas other services such as a dietician or physical therapy are freely accessible. A second limitation could be the retrospective nature of the data collection. The data does however correspond to the real world situation of referral from GP to OS. Finally, some diagnoses mentioned in the referral letters such as loose body, osteochondral lesion, effusion, chondrocalcinosis, Baker’s cyst, joint overuse or patella maltracking might in some cases be standalone diagnoses but in other cases might be manifestations of OA. This can lead to an underestimation of incidence of OA by GP’s when only these secondary manifestations are mentioned among the differential diagnosis. However, in the current study design a underestimation of such is inevitable as a matching diagnosis can only be determined when both the GP and the OS stated their diagnoses unambiguously in their differential diagnosis. We believe no underestimation of OA is present among OS diagnoses as before mentioned diagnoses were only stated as standalone diagnoses as no radiological sings of OA were present.

In conclusion, especially in younger patients there were differences in the (differential) diagnosis mentioned in referral letters of GP’s with the orthopedic diagnosis at the OPC. Also, in younger patients the frequency of matching diagnoses was evidently lower, possibly explained by a reduced awareness of the occurrence of OA at younger age among GP’s, leading to the underdiagnosing of OA in younger patients. Better recognition of OA in younger patients by increasing awareness on the prevalence of OA in younger patients might improve referral efficacy and can potentially improve standard of care. Referrals for atraumatic meniscal lesions in patients over 45 years old rarely led to surgery, making rapid referral for surgical consultation less necessary as the majority of patients is treated conservatively nowadays. In order to improve efficacy of referrals, future research should focus on the effectiveness of musculoskeletal triage, educational programs for patients on treatment options and selfcare for degenerative knee complaints and it should focus on improving referral guidelines and programs promoting the use conservative treatment modalities by GP’s as described in the SCS.

## Data Availability

The datasets used and/or analysed during the current study are available from the corresponding author on reasonable request.

## Abbreviations

OA: Osteoarthritis
GP: General practitioner
OPC: Outpatient clinic
SCS: Stepped care strategy
